# Helping Apo2L/TRAIL in the Battle: Synergistic Therapeutic Approaches for Cancer Therapies

**DOI:** 10.3390/ijms27167068

**Published:** 2026-08-07

**Authors:** Elena Valeria Fuior, Madalina Dumitrescu, Marius Gabriel Multescu, Bianca Sanziana Daraban, Madalin Ghinea, Oana Mirancea, George E. D. Petrescu, Felix Mircea Brehar, Ana Maria Vacaru, Radu Ionita, Violeta Georgeta Bivol, Irina Florina Tudorache, Andreea Popa, Ioana Madalina Fenyo, Evangelia Zvintzou, Anca Violeta Gafencu

**Affiliations:** 1 Institute of Cellular Biology and Pathology “Nicolae Simionescu”, Romanian Academy, 050568 Bucharest, Romania; elena.fuior@icbp.ro (E.V.F.); madalina.dumitrescu@icbp.ro (M.D.); marius.multescu@icbp.ro (M.G.M.); sanziana.daraban@icbp.ro (B.S.D.); madalin.ghinea@icbp.ro (M.G.); oana.mirancea@icbp.ro (O.M.); ana.vacaru@icbp.ro (A.M.V.); radu.ionita@icbp.ro (R.I.); violeta.bivol@icbp.ro (V.G.B.); irina.tudorache@icbp.ro (I.F.T.); andreea.popa@icbp.ro (A.P.); madalina.fenyo@icbp.ro (I.M.F.); lilia.zvintzou@gmail.com (E.Z.); 2 Neurosurgery Clinic, “Carol Davila” University of Medicine and Pharmacy, 020021 Bucharest, Romania; george.petrescu@umfcd.ro (G.E.D.P.); felix.brehar@umfcd.ro (F.M.B.); 3 Department of Neurosurgery, Bagdasar-Arseni Emergency Clinical Hospital, 041915 Bucharest, Romania; 4 Pharmacology Laboratory, Department of Medicine, University of Patras, 26504 Rio Achaias, Greece

**Keywords:** TRAIL, apo2L, death receptor, apoptosis, combinatorial therapy, cancer

## Abstract

Tumor necrosis factor-related apoptosis-inducing ligand (TRAIL) selectively triggers apoptosis in malignant, infected, or stressed cells while sparing normal tissues, making it an attractive therapeutic candidate. However, many tumors exhibit intrinsic or acquired resistance to TRAIL, driven by reduced DR4/DR5 surface expression, elevated decoy receptor levels, dysregulated DISC assembly, overexpression of c-FLIP and anti-apoptotic Bcl-2 family proteins, or activation of survival pathways such as NF-κB, PI3K/Akt, and MAPK. Moreover, TRAIL receptors can initiate non-canonical signaling pathways that promote migration, invasion, and metastasis in specific oncogenic contexts, thereby further limiting therapeutic efficacy. We aimed to integrate mechanistic insights into TRAIL biology with current therapeutic advances, providing a comprehensive framework for understanding resistance and for designing rational TRAIL-based combination strategies. We summarized the structural and signaling features of TRAIL receptors, outlined the major determinants of TRAIL sensitivity, and evaluated predictive biomarkers that may guide patient selection. In addition, we examined next-generation TRAIL agonists and targeted delivery systems developed to enhance receptor clustering, pharmacokinetics, and tumor specificity. Together, these insights highlight the therapeutic promise of mechanistically informed TRAIL combinations. A deeper understanding of resistance pathways and biomarker-guided stratification will be essential for restoring apoptotic competence and improving clinical outcomes.

## 1. Introduction

Tumor necrosis factor-related apoptosis-inducing ligand (TRAIL/Apo2L/TNFSF10/CD253) is a member of the TNF-ligand superfamily [1,2]. TRAIL is a key immunoregulatory molecule with proapoptotic potential, expressed by various immune cell types, and plays a key role in regulating immune responses under normal conditions and in diseases. TRAIL contributes to immune surveillance by enabling NK cells and activated CD8^+^ T cells to induce apoptosis in stressed, infected, or transformed cells through engagement of TRAIL death receptors [3,4]. Liver-resident NK cells can express TRAIL constitutively, whereas most circulating NK and T cells upregulate TRAIL only after inflammatory activation [3,4].

A defining biological property that initially positioned TRAIL as a highly promising anticancer agent is its ability to selectively induce apoptosis in cancerous, infected, or transformed cells, as well as in activated immune cells, while sparing normal, healthy tissues from cytotoxic effects [5]. This selective feature of TRAIL makes it an attractive molecule for cancer treatment, and numerous clinical trials have been initiated; however, the intrinsic or acquired resistance of many cancer cells to TRAIL-induced apoptosis, together with the limited cytotoxic efficiency observed in certain tumor types, has driven the search for adjuvant strategies and rational combination approaches [6,7,8]. Identifying TRAIL sensitizers in cancer cells, empirically or by rational design, that promote TRAIL-induced apoptosis remains an exciting area of cancer treatment research.

This review summarizes the structural and signaling features of TRAIL and its receptors, examines the key mechanisms underlying intrinsic and acquired resistance, outlines emerging predictive biomarkers relevant for patient stratification, and integrates these mechanistic insights with current therapeutic advances, highlighting next-generation TRAIL agonists and the diverse combination strategies designed to restore apoptotic sensitivity across tumor types.

## 2. TRAIL Protein Structure, Receptors, and Apoptotic Function

### 2.1. TRAIL Isoforms and Structural Features

In humans, at least three isoforms of TRAIL were identified: TRAIL-α (GenBank: NP_003801.1, full length, 281 amino acids), TRAIL-β (mRNA lacks exon 3, 119 amino acids), and TRAIL-γ (mRNA lacks exons 2 and 3, 98 amino acids), as shown in Table 1. The loss of exon 3 introduces a frameshift, so the extracellular domains of TRAIL-β and TRAIL-γ differ from those of TRAIL-α; consequently, TRAILβ and TRAILγ isoforms do not induce apoptosis [9]. Several alternatively spliced and truncated TRAIL variants exhibited distinct activities in NF-κB activation [10]. TRAIL-α protein (32 kDa) was identified based on sequence homology with TNFα and FasL [1]. Interestingly, in murine cells, only the full-length TRAIL has been identified. Murine TRAIL has 291 amino acids and shares ~65% identity with the human homolog [1].

TRAIL is a type II transmembrane glycoprotein whose extracellular region assembles into a bell-shaped homotrimer, characteristic of TNF family ligands [2]. A distinctive structural feature of TRAIL is the Zn^2+^ ion located at the trimer interface, which is essential for maintaining trimer stability and apoptotic function; substitution of Cys230 with alanine or serine markedly impairs TRAIL-induced apoptosis [11].

TRAIL can be found anchored in the cell membrane, and it can be proteolytically cleaved by cathepsin E to generate a 21 kDa soluble fragment with apoptotic potential [12]. TRAIL homotrimer binds to trimerized receptor molecules [13,14]. To exert cytotoxic activity, a 12-16-amino-acid insertion loop from the TRAIL extracellular domain occupies the receptor-binding site. It alters the common receptor-binding surface, as demonstrated by mutagenesis studies [13].

### 2.2. TRAIL Receptors and Their Signaling Roles

**Figure 1:**
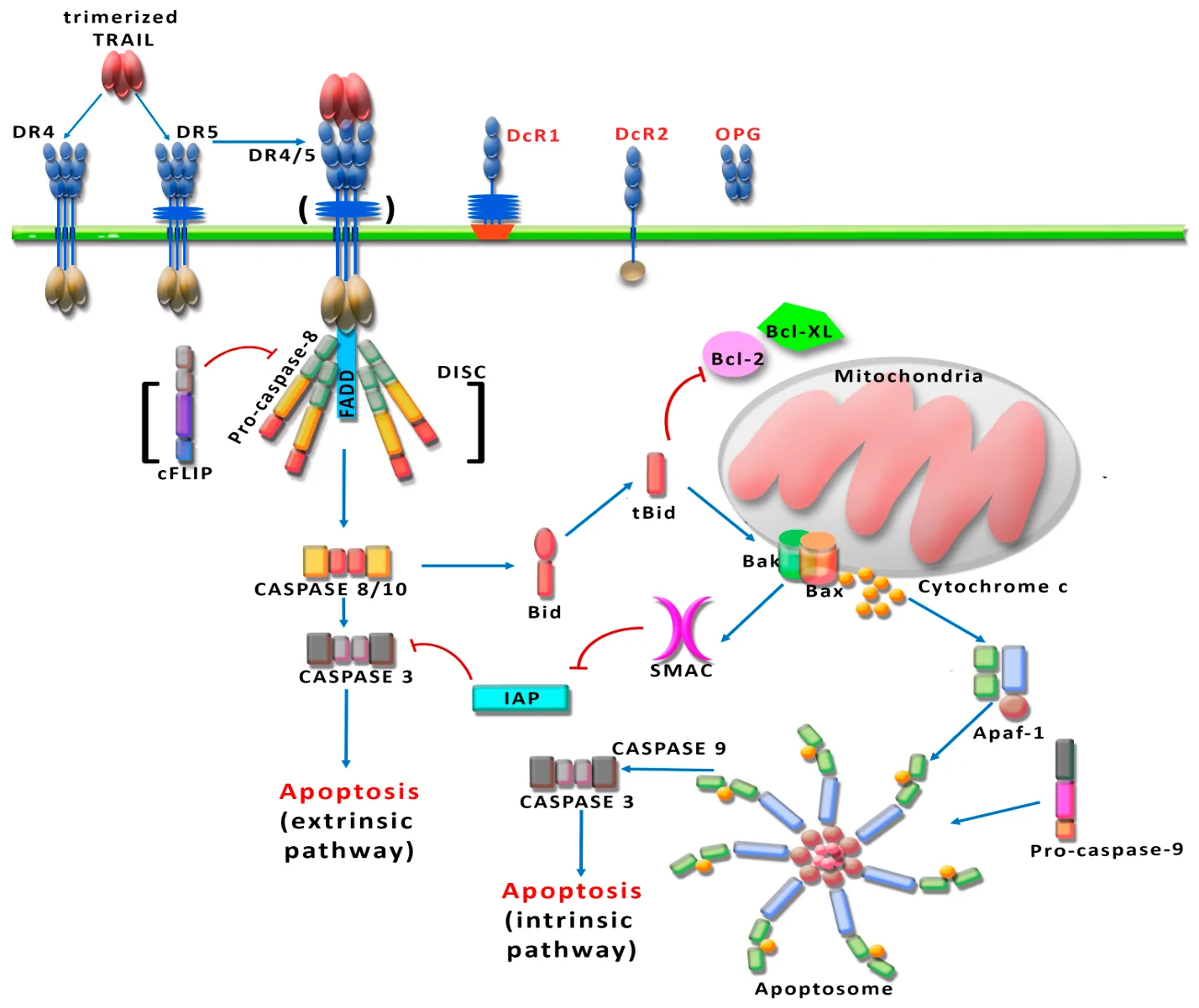
TRAIL receptors and the mechanism of TRAIL-induced apoptosis. In humans, DR4 and DR5 are functional in inducing apoptosis, whereas DcR1, DcR2, and Osteoprotegerin (OPG) inhibit TRAIL-induced apoptosis. After cleavage and trimerization, TRAIL binds to trimerized DR4 or DR5, inducing DISC formation and activating Caspase 8 or 10, which in turn activates Caspase 3 in the extrinsic apoptosis pathway. The intrinsic pathway involves the activation of Bid, mitochondrial release of cytochrome c, and the formation of the apoptosome, composed of Apaf-1, cytochrome c, and procaspase-9, which assembles into a heptameric activation complex that initiates the caspase cascade.

To fulfill various biological functions, human TRAIL interacts with four receptors found on the cell surface: DR4/TRAIL-R1, DR5/TRAIL-R2, DcR1/TRAIL-R3, and DcR2/TRAIL-R4 [15,16,17,18] or with the soluble TRAIL receptor, Osteoprotegerin (OPG) [19], as illustrated in Figure 1.

Binding to a certain receptor type dictates different TRAIL functions, as summarized in Table 2. DR4 and DR5 receptors are type I transmembrane proteins characterized by the presence of extracellular cysteine-rich domains, a transmembrane region, and an intracellular domain of 80 amino acids called “death domain” [20], and differ in the small extracellular region, which confers to DR5 a higher flexibility resulting in more efficient ligand-induced trimerization and consequently in a higher affinity for TRAIL. This death domain plays a crucial role in transducing apoptotic signaling and serves as the death adaptor protein [21,22,23]; thus, its interaction with the DR4 and DR5 receptors triggers apoptosis. Decoy receptors DcR1 and DcR2 inhibit TRAIL-induced apoptosis because they lack a functional death domain: DcR1 is bound to the cell surface and lacks the intracellular death domain [24,25,26], whereas DcR2 is a transmembrane protein with a nonfunctional death domain [27,28]. DcR1 and DcR2 attenuate TRAIL-induced apoptosis by sequestering TRAIL away from DR4/DR5 or by integrating into non-productive mixed receptor complexes that block DISC assembly [25,29]. Osteoprotegerin (OPG), present mainly as a soluble disulfide-linked dimer, suppresses TRAIL-mediated apoptosis, conferring survival advantages on cancer cells and inhibiting osteoclast differentiation and bone resorption [30,31,32]. 

In mice, there is only one TRAIL receptor with a functional death domain (mDR5), resembling human DR4/TRAIL-R1 and DR5/TRAIL-R2 [33].

### 2.3. Canonical TRAIL Signaling (Extrinsic and Intrinsic Pathways)

Apoptosis is a highly regulated form of programmed cell death essential for tissue homeostasis. It proceeds through either the extrinsic or intrinsic pathway, shown in Figure 1. In the extrinsic pathway, apoptosis is initiated by extracellular ligands such as TRAIL, which bind to their cognate death receptors (DRs) on the cell surface. Ligand engagement induces conformational changes in both the extracellular and intracellular death domains of the receptors, enabling recruitment of the adaptor protein FADD and subsequent association with procaspase-8 or -10 [23]. This assembly forms the death-inducing signaling complex (DISC), which also incorporates the inhibitory protein c-FLIP, a competitor of procaspase-8 at the FADD interface [34]. Caspase-8 undergoes autocatalytic activation at the DISC and is released into the cytoplasm, where it cleaves and activates the executioner caspases-3, -6, and -7, thereby initiating apoptosis through the canonical TRAIL pathway [35]. Caspase-8 also links the extrinsic and intrinsic pathways by cleaving Bid to tBid, thereby antagonizing Bcl-2 and Bcl-xL and activating Bax and Bak [36]. Bax/Bak oligomerization permeabilizes the mitochondrial outer membrane, enabling the release of cytochrome c, Smac, endonuclease G, and AIF. In the cytosol, cytochrome c associates with Apaf-1, thereby activating Apaf-1 and promoting apoptosome assembly—a complex composed of seven Apaf-1 and seven cytochrome c molecules—which activates procaspase-9 [37,38]. Caspase-9 then activates executioner caspases, driving the proteolytic events characteristic of apoptosis [39]. Smac facilitates this process by neutralizing IAPs [40], while AIF and endonuclease G translocate to the nucleus to promote chromatin condensation and DNA fragmentation [41].

### 2.4. Non-Canonical TRAIL Signaling and Context-Dependent Outcomes

Engagement of TRAIL receptors, particularly DR5, can also initiate non-canonical signaling pathways that modulate survival, migration, inflammation, and metastatic potential. Among the most important, NF-κB cascade, which may induce anti-apoptotic genes such as c-FLIP [42]; the MAPK pathways activated through a secondary RIP1–TRAF2–NEMO complex formed downstream of the DISC, driving JNK, p38, and IKK activation [43]; the PI3K/Akt axis, which enhances cell survival and contributes to TRAIL resistance; and the RhoA/Rac1 signaling, which drives cytoskeletal remodeling, migration, and invasion [44]. In specific cellular contexts, DR4 can engage UPR-associated ER stress pathways, and DR5 can mediate TRAIL-induced cell motility [45].

Components of the DR5/FADD/caspase-8 death pathway, traditionally linked to apoptosis, can paradoxically promote cancer progression, and high caspase-8 and DR5 expression correlate with lymph node metastasis and poor patient survival [46]. By contrast, the deficiency of the apoptotic TRAIL receptor in a murine model does not affect primary tumor formation. Still, it markedly increases lymph node metastasis, demonstrating that TRAIL receptor functions as a metastasis suppressor [47].

Several tumor-intrinsic factors determine whether TRAIL receptor engagement triggers apoptosis or instead diverts signaling toward non-canonical, pro-survival outputs. Cancer stem cells, known for their resistance and metastatic potential, express insufficient levels of active caspase-8, leading to incomplete DISC assembly, and overexpress Bcl-2, thereby shifting TRAIL signaling toward survival-promoting pathways [48]. Elevated c-FLIP further suppresses caspase-8 activation and consequently inhibits TRAIL-induced apoptosis [49]. Specific oncogenic contexts—such as KRAS mutations [44], p53 loss or mutation, or heightened ER stress [45]—reinforce this bias toward non-apoptotic signaling. In such settings, non-canonical TRAIL signaling, in which TRAIL receptors promote tumor progression rather than cell death, becomes a key determinant of cancer stem cell resistance and aggressiveness.

## 3. General Considerations on TRAIL-Based Therapies

TRAIL-based therapy has been tested for various malignancies. Glioblastoma cell lines express DR4 and DR5 receptors and therefore are susceptible to TRAIL-induced apoptosis [50]. The TRAIL–DR5 system also plays an essential role as a transducer of pro-inflammatory and angiogenic signals in human brain tumors [51] and in other cancers, such as non-small cell lung carcinoma [52]. Although early studies were encouraging, later investigations revealed substantial variability in glioblastoma sensitivity to TRAIL-induced apoptosis [51]. In acute leukemia, TRAIL suppresses the growth of early myelodysplastic progenitors without affecting normal hematopoiesis, as human and murine hematopoietic progenitors are insensitive to TNF-family receptor-mediated apoptosis [53,54]. Numerous clinical trials have been conducted using TRAIL-based therapeutics [55,56,57]. Dulanermin (AMG-951) is a non-tagged recombinant TRAIL protein advanced to phase III clinical trials [58,59]. Still, despite promising preclinical results, it did not demonstrate significant therapeutic benefit [60]: a synthetically cross-linked version, Apo2L.XL exhibited markedly improved pro-apoptotic activity, reducing the viability of 257 cell lines from a panel of 479, compared with only 146 cell lines responding to plain Dulanermin [61]. Aponermin, another recombinant human TRAIL engineered for enhanced stability and improved DR4/DR5 activation, was recently approved in China for the treatment of refractory multiple myeloma, administered in combination with thalidomide and dexamethasone after at least two prior systemic therapies [62,63].

Poor pharmacokinetics and intrinsic or acquired resistance limit the efficacy of recombinant TRAIL. To address these challenges, improved TRAIL molecules have been developed. Single-chain TRAIL (scTRAIL), generated by joining three extracellular fragments of TRAIL with peptide linkers, forms optimized trimers and reduces nonspecific monomer aggregation. Additional recombinant variants, such as leucine-zipper TRAIL (LZ-TRAIL) [64,65] and isoleucine-zipper-tagged (Ilz-TRAIL) [66], enhance oligomerization and cytotoxicity. Another strategy fuses TRAIL to the TWEAK receptor Fn14, producing a highly efficient TRAIL analog [67]. TWEAK, an abundant cytokine found in the tumor environment, further oligomerizes TRAIL receptors and increases the cytotoxic activity. The specificity can be improved by fusing scTRAIL to antibody fragments that target TRAIL to specific tumors [68].

Second- and third-generation TRAIL agonists are designed to overcome the limitations of earlier agents, such as weak multimerization, insufficient receptor clustering, rapid clearance, and resistance to apoptosis, thereby improving pharmacokinetics, enhancing tumor selectivity, and reducing off-target toxicity [69]. These include extremely diverse TRAIL formulations, such as recombinant proteins linked to scFv, targeting antibodies or peptides, or PEG-modified variants. Multivalent fusion proteins such as the bivalent APG-350 [70], the PEGylated, trimer-forming TLY012 [71,72], and the hexavalent eftozanermin alfa (ABBV-621/APG-880) [73] exemplify this approach. DR4/DR5-targeting antibodies include the bispecific, tetravalent antibody RG7386 (RO6874813) [74] and the third-generation tetravalent DR5 agonistic antibody INBRX-109, built on a camelid-derived single-domain antibody platform [75].

The tumor microenvironment contributes to resistance by modulating DR4/DR5 expression and function. Hypoxia, a hallmark of rapidly growing solid tumors, is associated with enhanced anaerobic glycolysis and leads to severe extracellular acidity within the tumor microenvironment. This persistent acidification may attenuate TRAIL-induced apoptosis pathways [76]. However, in acidic extracellular environments, TRAIL can also induce RIPK-1-dependent necroptosis, as shown in adeno- and hepatocarcinoma cells [77].

Systemic delivery of TRAIL faces challenges such as protein instability, intrinsic or acquired resistance to apoptosis, off-target toxicity, and poor availability at the tumor site. Targeted delivery systems enable TRAIL to induce apoptosis in tumor cells effectively, overcome these barriers, and improve therapeutic efficacy [78]. Local administration offers many advantages: intracranial or intratumoral delivery ensures direct exposure of tumor cells to TRAIL. Intratumoral injections (2 × 2 μg) of Apo2L/TRAIL eradicated intracranial human malignant U87 glioma xenografts in athymic mice, without toxicity [79]. To eradicate disseminated glioma cells, TRAIL-secreting neural stem cells were implanted into human glioblastoma xenografts, inducing apoptosis and significantly inhibiting tumor growth [80].

To extend TRAIL’s short half-life, diverse delivery platforms have been developed, including viral vectors [81], nanoparticles [82,83], and cell-based carriers such as platelets [84], mesenchymal stem cells (MSCs) [85,86], and MSC-derived vesicles [87].

Despite these therapeutic advances, TRAIL monotherapy has shown limited efficacy in many tumor types. This lack of durable clinical responses is largely attributable to widespread intrinsic and acquired resistance, which is addressed in the following section.

## 4. Mechanisms of TRAIL Resistance

The limited efficacy of TRAIL monotherapy across many tumor types reflects the complex molecular mechanisms through which cancer cells evade TRAIL-induced apoptosis. These resistance pathways affect receptor availability, DISC assembly, apoptotic signaling, and compensatory survival networks, collectively diminishing the therapeutic potential of TRAIL. Although TRAIL is an attractive anticancer agent, many human tumors exhibit poor responsiveness to recombinant soluble TRAIL or other TRAIL receptor agonists [68]. This phenotype arises from both constitutive resistance in aggressive malignancies and acquired resistance that develops following repeated TRAIL exposure, and involves alterations in death receptor expression, intracellular signaling networks, and receptor trafficking.

Intrinsic resistance frequently results from low surface availability of DR5, aberrant DR5 internalization, or improper intracellular localization, and does not support efficient DISC formation [88]. As a result, even high concentrations of TRAIL fail to trigger a robust apoptotic response, establishing a baseline resistance that limits therapeutic efficacy.

Acquired resistance emerges when tumor subclones that survive initial TRAIL exposure activate alternative signaling pathways that promote cell proliferation. Persistent stimulation of NF-κB, PI3K/Akt, and MAPK/JNK enables these cells to withstand subsequent TRAIL treatments, a phenomenon often described as “fractional survival” [89]. Over time, this adaptive signaling rewires the apoptotic machinery, progressively reducing TRAIL sensitivity. Importantly, key apoptotic proteins such as FADD and procaspase-8 participate in both death and survival signaling pathways, allowing tumor cells to remodel downstream pathways in ways that generate cross-resistance to combination therapies, even when multiple agents are used simultaneously [89].

TRAIL-based therapies also intersect with immune checkpoint pathways, contributing to TRAIL resistance. The mechanism of this process involves TRAIL-induced upregulation of PD-L1, a key immune checkpoint that tumors exploit to suppress T-cell activity [90,91]. This effect was demonstrated in esophageal squamous cell carcinomas [90], triple-negative breast cancer cells [92], and gastric cancer [93]. PD-L1 promotes resistance to TRAIL-mediated apoptosis by binding and activating EGFR [93], thereby creating a vicious cycle.

To overcome these barriers, next-generation TRAIL receptor agonists, including DR5-specific variants and agonistic antibodies, have been developed to improve receptor clustering, enhance apoptotic signaling, and bypass the limitations of first-generation soluble TRAIL formulations [69]. The mechanisms of apoptosis resistance in specific tumor types have been comprehensively reviewed in [94].

Together, the intrinsic and acquired resistance mechanisms highlight the need for reliable predictive biomarkers to identify tumors with preserved apoptotic competence and to guide the rational design of combination strategies to restore TRAIL sensitivity.

## 5. Predictive Biomarkers for TRAIL Sensitivity

In precision oncology, prognostic biomarkers predict the course of the disease, while predictive biomarkers indicate which patients are most likely to respond to or experience adverse effects from particular therapies. Given the heterogeneity of TRAIL responsiveness across tumor types, predictive biomarkers are essential for stratifying patients and for identifying molecular contexts in which TRAIL-based therapies or combinations are most likely to succeed. Identifying biomarkers for treatment response prediction is thus of foremost importance. To address their clinical translatability, these emerging indicators must be distinguished by their feasibility of detection alongside their clinical limitations.

### 5.1. Clinically Detectable Assays and Biomarkers

Several molecular signatures can be monitored using standard clinical laboratory platforms, such as quantitative PCR (qPCR), immunohistochemistry (IHC), or transcriptomic microarrays.

**Death/Decoy Receptor Ratios** (qPCR/IHC Assay): High cell-surface expression of functional death receptors (DR4, DR5) coupled with reduced levels of TRAIL decoy receptors (DcR1, DcR2) is a strong positive predictor of TRAIL sensitivity. Specifically, a quantitative mRNA level ratio of TRAIL-R1/(TRAIL-R3 + TRAIL-R4) < 0.85 serves as a measurable threshold predicting TRAIL resistance [95].

**Transcriptomic Networks** (Gene Expression Profiling): Genome-wide mRNA expression profiles from patient biopsies can identify responsive profiles. A validated 71-gene signature—dominated by interferon (IFN)-induced genes and major histocompatibility complex (MHC) genes—accurately predicts high TRAIL sensitivity across extensive human cancer cell line panels [96]. Clinically, treating tumors with IFN-γ can actively augment TRAIL-induced apoptosis by priming the extrinsic apoptotic cascade.

**Hyperactivated Kinases** (Phospho-Specific IHC): Constitutive activation of downstream compensatory hubs, such as the PI3K/Akt pathway, serves as a measurable negative predictive biomarker for TRAIL monotherapy [97]. When these pathways are modulated pharmacologically, tumors exhibit a strong, trackable synergistic sensitivity to TRAIL combinations. Similarly, high baseline STAT3 activity can be quantified; its subsequent inactivation (e.g., via tunicamycin) significantly potentiates TRAIL-induced cell death [98].

### 5.2. Preclinical and Experimental Molecular Indicators

Certain biomarkers provide invaluable mechanistic insights but currently rely on complex assays primarily restricted to specialized research settings rather than routine clinical pathology.

**Cell Surface Glycosylation Patterns.** The level of core fucosylation on cell-surface proteins regulates TRAIL receptor clustering. High fucosylation is a negative predictor for TRAIL combinations, whereas defucosylation, achieved by knocking down the FUT8 gene encoding α(1,6)- fucosyltransferase, sensitizes resistant tumors [99]. Additionally, GALNT14-mediated O-glycosylation of DR4 and DR5 serves as an experimental indicator that promotes receptor clustering and subsequent apoptosis [100].

**Intracellular Apoptotic Rheostat.** The ratio of pro- and anti-apoptotic proteins in the cytoplasm determines the final decision to die. High baseline expression of inhibitors such as c-FLIP [101], IAPs [102], or Bcl-2 [103] acts as a molecular blocker of TRAIL-induced apoptosis. Conversely, mutations or modulators that downregulate the gene expression of these proteins can serve as predictive signatures of a restored apoptotic response, as demonstrated by Bcl-xL inhibitors (ABT-263 and ABT-737) that sensitized human pancreatic cancer cells to TRAIL [104].

**Transcriptional Stress Sensors.** Intracellular stress responses, such as NF-κB signaling, play a highly complex role in TRAIL sensitivity [105]. Moreover, transient upregulation of transcription factors such as CHOP (induced experimentally by oligomycin A or other agents) drives DR5 expression, thereby enhancing the overall apoptotic effect [106].

### 5.3. Clinical Limitations and Implementation Barriers

Despite their predictive potential, transitioning these molecular indicators into routine oncological practice faces major biological and technical obstacles:

**Spatial and Temporal Heterogeneity:** Tumor biopsies often do not reflect the actual expression of death and decoy receptors, which vary across regions within the same solid tumor. Furthermore, receptor expression changes over time during chemotherapy, so a single baseline biopsy is a poor predictor of long-term response.

**Lack of Standardized Cutoff Thresholds:** Beyond specific experimental models (such as the ratio TRAIL-R1/(TRAIL-R3 + TRAIL-R4) < 0.85), there are no clinically validated cutoff thresholds for distinguishing “TRAIL-positive” from “TRAIL-resistant” tumors using standard clinical IHC.

**Assay Standardization:** Measuring complex post-translational modifications, such as cell-surface GALNT14 O-glycosylation or core fucosylation, requires advanced mass spectrometry or specialized lectin-binding assays, which are lacking in standard hospital pathology laboratories.

**Signaling Redundancy:** Because TRAIL resistance can act at multiple levels, evaluating a single biomarker in isolation often yields false-positive predictions. Clinical success will likely necessitate integration of multiple markers.

## 6. Combinatorial Therapeutic Strategies Based on TRAIL

Building on the mechanistic insights into TRAIL resistance and the emerging predictive biomarkers, numerous agents have been developed to sensitize tumor cells to TRAIL-induced apoptosis. Although these compounds belong to diverse chemical classes, they frequently act through shared molecular mechanisms that counteract intrinsic or acquired resistance. These combinatorial strategies target distinct nodes of the resistance network and aim to restore or amplify apoptotic competence across diverse tumor types. To provide a clearer mechanistic perspective, we focus on the mechanistic nodes targeted by TRAIL-sensitizing strategies: (1) modulation of death receptor availability, (2) enhancement of DISC assembly, (3) inhibition of anti-apoptotic proteins, (4) regulation of survival pathways, induction of ER stress/ROS signaling, and (5) attenuation of noncanonical TRAIL outputs, while illustrating each mechanism with representative compounds from different classes. Importantly, a single compound can engage several of these nodes simultaneously rather than acting through a single mechanism.

### 6.1. Increasing DR4/DR5 Surface Availability and Reducing DcR1/DcR2 Interference

A major mechanism of TRAIL resistance is the low expression of DR4/DR5 and the high expression of decoy receptors (DcR1/DcR2). Numerous agents restore TRAIL sensitivity by upregulating functional death receptors, activating ER stress pathways, generating ROS, or reducing decoy receptors, thereby enabling efficient DISC formation and caspase-8 activation.

**ER stress-driven DR5 induction.** This is the best-characterized and most consistently reported route to death receptor upregulation. Quercetin enhanced DR4/DR5 through CHOP activation under ER stress [107], and separately reduced c-FLIP [108,109] and promoted Mcl-1 degradation [110]. Ursolic acid [111] and tanshinone IIA [112] both induced DR5 through the PERK/ATF4 axis, with tanshinone IIA additionally suppressing survivin via STAT3 inhibition. Artesunate triggered ER stress and selectively upregulated DR5, synergizing with TRAIL both in vitro and in vivo [113]. Among antibiotic-class agents acting through the same node, monensin induced ER stress and reduced c-FLIP [114], salinomycin boosted apoptosis in TRAIL-resistant glioblastoma cells by upregulating DR5 [115], and tunicamycin, a classical ER stress inducer, enhanced TRAIL-induced apoptosis by upregulating DR5 while sparing normal peripheral blood mononuclear cells [116].

**ROS-dependent receptor** induction. This pathway signals upstream of or in parallel with ER stress. Curcumin induced ROS-dependent DR5 upregulation together with IAP reduction [117], and dihydroartemisinin induced ROS alongside DR5 and other apoptotic proteins [118]. EGCG likewise activated DR4/DR5 in colon, hepatocellular, and melanoma cells [119,120,121], in this case combined with NF-κB inhibition [122], reduction in FLIP/Mcl-1/Bcl-2 [123], and decreased Bid [124]. The proteasome inhibitor bortezomib induced DR5 via ROS, mitochondrial depolarization, caspase activation, and suppression of Bcl-2/Bcl-xL [125].

**Transcription factor- and epigenetic-level regulation of DR4/DR5.** Apigenin increased DR5 via ANT2 inhibition [126], activated p53-dependent DR4/DR5 expression [127], and modulated DR5/c-FLIP splicing [128], making it one of the few agents shown to act at the level of receptor mRNA processing rather than only transcription. Periplocin synergized with TRAIL through a distinct transcriptional route, downregulating the transcription factor FoxP3 to increase DR4/5, alongside caspase-3/FADD activation and survivin reduction [129]. Gene-editing approaches directly confirm this node: CRISPR-mediated Klotho upregulation [130] and miR-133 knockdown [131] both increased DR4/DR5 and apoptosis.

**Decoy receptor suppression.** Subtoxic-dose cisplatin enhanced TRAIL-mediated apoptosis by downregulating DcR2 [132]. Knocking down adenine nucleotide translocase-2 (ANT2) sensitized cells to TRAIL by simultaneously increasing DR4/DR5 expression and downregulating DcR2 [133], while direct DcR2 silencing resensitized resistant breast cancer cells to TRAIL [134]. These three findings converge on decoy receptor suppression as a mechanistically distinct, and comparatively less explored, alternative to death receptor upregulation.

**Cytokine-mediated receptor induction.** Interleukin-6 increased DR4/5 expression and activated caspases 3, 8, and 9 across several cancer cell lines [135]. Interferon-β upregulated DR4, DR5, and caspase-3 in melanoma cells [136], and the same cytokine enhanced TRAIL-induced apoptosis in glioblastoma cells through DR5 induction [137].

**Cytoskeletal disruption and DNA damage through chemotherapy.** A broad range of conventional cytotoxic agents also upregulate DR4/DR5, generally without a fully resolved upstream mechanism. Colchicine, a microtubule disruptor, increased DR5 expression and activated caspase-3 [138]. Vinca alkaloids, etoposide, adriamycin, and camptothecin similarly upregulated DR4 and DR5 [139], as did paclitaxel in prostate cancer cells [140], mitoxantrone [141], and the thymidylate synthase inhibitor trifluorothymidine, which additionally reduced cFLIP and XIAP while increasing Bax [142]. Thymoquinone upregulated DR4/DR5 while also directly enhancing TRAIL binding to its cell-surface receptors, a distinct route from transcriptional induction alone [143,144]. Two agents, daurisoline [145] and theophylline [146], sensitized breast cancer cells by upregulating DR5, but their upstream mechanism has not yet been characterized; this remains a gap worth flagging rather than assuming these agents fit neatly into one of the mechanistic nodes above.

### 6.2. Modulating Intracellular Apoptotic Signaling

TRAIL resistance frequently arises from blockades in the apoptotic cascade, driven by elevated levels of cFLIP, survivin, Bcl-2/Bcl-xL, XIAP, cIAPs, or insufficient caspase activation. Agents restoring TRAIL sensitivity through this route act at three distinct points of the intracellular cascade: direct suppression of anti-apoptotic regulators, mitochondrial priming via Bcl-2/Bcl-xL inhibition, and IAP neutralization combined with cell-cycle arrest.

**Suppression of cFLIP, survivin, and IAPs.** Low-dose YM155, in synergy with TRAIL, enhances apoptosis in HeLa cells by downregulating cFLIP and survivin [147]. Cisplatin enhanced apoptosis by upregulating caspase-8, caspase-9, and Bax [132] or by a p53-dependent mechanism [148]. Obatoclax, a BH3 mimetic, reduced XIAP levels and promoted activation of caspases 3, 8 and 9, and also upregulated DR4 and DR5 levels [149]. Decursin, an anti-tumoral pyranocoumarin, increases production of ROS in NSCLC, leading to subsequent activation of the ER stress pathway PERK/ATF4, increased expression of DR5, and decreased levels of survivin and Bcl-xL [150]. J7, a methyl jasmonate analog, potentiated TRAIL-induced apoptosis in HepG2 cells via ROS-mediated caspase pathways, including down-regulation of XIAP, cIAP-1, and Bcl-xL, activation of caspases, and cleavage of poly (ADP-ribose) polymerase and phospholipase γ-1 [151]. Compound C, a tiopyrrazolopyrimidine derivative, enhanced TRAIL-induced apoptosis by ROS-mediated down-regulation of c-FLIP(L) and Mcl-1 [152]. Hydroxychavicol, a polyphenol from Piper betel leaf, downregulates the expression of the antiapoptotic proteins FLIP and XIAP, a process mediated by ROS [153].

**Bcl-2/Bcl-xL inhibition and mitochondrial priming.** Direct inhibition of Bcl-2 family proteins restores mitochondrial engagement. The Bcl-2 inhibitor venetoclax synergized with TRAIL in inducing apoptosis in TP53INP2-positive acute myeloid leukemia [154]. Inhibition of Bcl-2 with venetoclax/ABT-199 overcame resistance in glioblastoma cells [155]. Combined treatment of urothelial carcinoma of the bladder with TRAIL and low-dose gemcitabine enhanced apoptosis by Bcl-2 inhibition and caspase activation [156]. Administration of TRAIL and an anti-tumoral sialic acid-binding lectin from the oocytes of Rana catesbeiana exhibited a synergistic apoptosis-inducing effect in malignant mesothelioma by significantly increasing the level of proapoptotic Bim, which translocates to the mitochondria, causing the release of cytochrome c [157]. Mitomycin C synergized with adenovirally expressed TRAIL and mild hyperthermia, triggering mitochondria-dependent apoptosis through JNK-mediated Bcl-xL phosphorylation and Bak activation [158].

**Smac mimetics and CDK inhibitors.** Smac mimetics neutralize IAPs and strongly amplify TRAIL signaling. Adenoviral co-expression of TRAIL and Smac, in combination with SNS-032, a cyclin-dependent kinase (CDK) inhibitor, synergized to enhance anti-tumoral activity in pancreatic cells, stimulating apoptosis by down-regulation of protein expression for CDKs-2, -7, and -9, and XIAP, Mcl-1, and Bcl-2 [159]. CDK9 inhibitor Atuveciclib (BAY 1143572) in combination with TRAIL sensitized pancreatic ductal adenocarcinoma cancer cells to TRAIL-induced cell death [160]. A more sophisticated approach involves using TRAIL and Smac with the oncolytic virus ZD55 and delivering it to IAP-rich hepatocellular carcinoma; moreover, in vivo experiments in mice showed that the combined treatment led to complete tumor regression in all animals due to a strong induction of apoptosis [161].

### 6.3. Inhibiting Compensatory Survival Pathways

In addition to apoptosis, TRAIL can activate pro-survival and pro-proliferative pathways, including PI3K/Akt, ERK, and transcription factors such as NF-κB and PPARs, which contribute to tumor invasion and metastasis [162], and thus, these pathways are targets for combinatorial therapies.

**PI3K/Akt.** Enhanced TRAIL apoptosis was reported for wortmannin in urothelial carcinoma of the bladder [156], matairesinol in LNCaP cells [163], and triciribine in PC-3 human prostate carcinoma cells [164]. The dual PI3K/mTOR inhibitor BEZ235, combined with TRAIL, produces strong synergistic antitumor activity across melanoma cell lines by modulating key regulators of both extrinsic and intrinsic apoptotic pathways—including c-FLIP, Bim, Bax, clusterin, Mcl-1, and members of the IAP family—and by inducing mitochondrial depolarization [165].

**MEK/ERK.** MEK1/2 inhibitor AZD6244 synergizes with TRAIL in melanoma cells [165]. The combinatorial treatment with the MEK inhibitor CH5126766 and statins upregulated TRAIL, inhibited the mevalonate pathway, and induced apoptosis in human breast cancer cells [166]. Interferon α (IFN-α) synergized with TRAIL in inducing apoptosis in renal cell carcinoma via ERK signaling [167].

**JAK/STAT.** Dovitinib, a multiple tyrosine kinase inhibitor, sensitizes resistant hepatocellular carcinoma cells to TRAIL-induced apoptosis by down-regulating phospho-STAT3 (Tyr705) (p-STAT3) and subsequently reducing the protein levels of STAT3-regulated proteins, Mcl-1, survivin, and cyclin D1, in TRAIL-treated cells. Combined treatment with TRAIL and dovitinib increased SHP-1 activity, a STAT3 inhibitor. In vivo, the combination of the anti-DR5 antibody tigatuzumab and dovitinib inhibited the growth of Huh-7 xenografts [168]. Another multikinase inhibitor, Sorafenib, synergized with TRAIL to induce apoptosis in the 8505C thyroid cancer cell line via the Jak-Stat3-Mcl1 pathway [169]. A selective JAK2 inhibitor, tyrphostin (AG-490), increases apoptosis in Jurkat T cell leukemia by suppression of Stat3 phosphorylation and downregulation of cIAP-1 and cIAP-2 [170].

**Rho/ROCK.** In osteosarcoma, TRAIL resistance was associated with the expression level of Rho-associated coiled-coil-forming protein kinase 2 (ROCK2), and ROCK2 silencing triggered the TRAIL-mediated apoptotic pathway and induced osteosarcoma cell death in vivo [171].

**PIM kinase.** PIM Ser/Thr kinases are oncogenes, and their overexpression in cancers leads to poor prognosis. Inhibition of PIM kinases enhanced TRAIL-mediated apoptosis in glioblastoma cells [172]. PIM knockdown and p62/SQSTM1 silencing led to DR5 overexpression, demonstrating the role of p62/SQSTM1 in mediating TRAIL-induced apoptosis [172].

**AMPK.** AMPK functions as a metabolic rheostat influencing TRAIL sensitivity. Treatment with compound C (an AMPK inhibitor), together with mesenchymal stem cells secreting TRAIL, increases apoptosis in glioblastoma, both in vitro and in vivo [173]. Conversely, AMPK activation by thapsigargin sensitizes esophageal cancer cells to TRAIL-induced apoptosis [174].

**NF-κB.** Combinatorial treatment with NF-κB inhibitors and TRAIL can reverse resistance and reduce tumor growth [24]. Casein kinase 2 (CK2) regulates TRAIL sensitivity in prostate cancer; CK2 inhibition combined with TRAIL suppresses p65 phosphorylation at Ser536, reduces nuclear translocation and transcriptional activity of NF-κB, and decreases expression of downstream anti-apoptotic genes [175]. In colorectal cancer, reducing NF-κB-dependent Bcl-xL expression, either by disrupting the IKK complex or through sulindac sulfide, potentiates TRAIL-induced apoptosis [176]. Combined adenoviral expression of TRAIL (d5hTRAIL) and IKK inhibition (AdIKKβKA) significantly increases apoptosis in A549 cells [177]. In MCF7 breast cancer cells, DcR2 overexpression drives TRAIL resistance, and both IKK inhibition and DcR2 silencing resensitize cells to adenoviral TRAIL [134]. Metformin, combined with TRAIL, induces colorectal cancer cell death in vivo by inhibiting AKT and NF-κB signaling and activating CHOP via ER stress [178].

**PPAR.** Troglitazone synergizes with TRAIL and induces apoptosis in TRAIL-resistant cancer cells [179]. Combinatorial treatment with TRAIL and troglitazone, a PPARγ ligand, induced apoptosis in TRAIL-resistant cancer cells by inhibiting GSK3β through early Akt-mediated Ser9 phosphorylation, followed by a strong transcriptional down-regulation of GSK3β expression, overcoming TRAIL-resistance [180]. Rosiglitazone, another PPARγ ligand, upregulated DR5 expression. Yet, the increase in TRAIL-mediated apoptosis in the presence of rosiglitazone is PPARγ-independent and instead involves a post-translational mechanism that leads to decreased expression of β-catenin [181] and c-FLIPs [182].

**YY1.** This transcription factor represses DR5 expression and contributes to TRAIL resistance. Rituximab, an anti-CD20 monoclonal antibody, sensitizes TRAIL-resistant B-NHL cells by inhibiting YY1, thereby restoring DR5 expression and apoptotic responsiveness [183].

**Nucleotide synthesis and DNA replication.** Irinotecan (CPT-11), an inhibitor of DNA Topoisomerase I, increased the expression of PCNA and activated caspase 3 [184]. Doxorubicin, a drug that intercalates into DNA and inhibits topoisomerase II [185], was shown to act synergistically with TRAIL in U1690 cells, and the underlying mechanisms involve mitochondrial dihydroorotate dehydrogenase and the pyrimidine pathway. Brequinar, a dihydroorotate dehydrogenase inhibitor, improves TRAIL sensitivity in U1690 cells and in other cell lines, with the most prominent effect observed in HT-29 colon cancer cells [186].

### 6.4. Epigenetic Regulation of TRAIL Sensitivity

Epigenetic regulation plays a central role in modulating TRAIL sensitivity, and inhibitors of histone deacetylases (HDACs) and histone methyltransferases are among the most effective classes of molecules that enhance TRAIL-induced apoptosis.

HDAC inhibitors act through multiple mechanisms, including upregulation of death receptors, suppression of anti-apoptotic proteins, restoration of caspase expression, and reinforcement of extrinsic–intrinsic apoptotic crosstalk. Among HDAC inhibitors, trichostatin A sensitized tumor cell lines to soluble TRAIL delivered by mesenchymal stem cells by upregulating death receptors and enhancing caspase-8 activation [187]. Valproic acid and ITF2357 potentiate TRAIL-induced apoptosis by a selective decrease in FLIP levels in hepatocellular carcinoma cells [188]. In the presence of interferon, valproic acid reestablished caspase-8 expression, thereby ensuring TRAIL signaling and a complete pathway for apoptosis induction [189]. Suberohydroxamic acid (SBHA) sensitized malignant mesothelioma cells to TRAIL apoptosis through down-regulation of the anti-apoptotic protein Bcl-xL [190]. LBH589 enhanced the TRAIL-induced apoptotic effect on endometrial cancer cells following metadherin knockdown [191]. Another potent HDAC inhibitor, MS-275, upregulated DR4/5, sensitizing TRAIL-resistant glioblastoma cells to stem cell-secreted TRAIL [192].

Histone methyltransferase inhibitors also play a significant role in overcoming TRAIL resistance. Chaetocin, an inhibitor of the histone methyltransferase SUV39H1, can overcome GBM cells’ resistance to TRAIL [193]. Chaetospirolactone, a fungal polyketide, shows strong promise in TRAIL-resistant pancreatic cancer cells by upregulating DR4 expression through epigenetic mechanisms involving inhibition of histone methyltransferases, thereby reversing apoptotic resistance [194].

### 6.5. Integrating Immune Checkpoint Inhibition into TRAIL-Based Therapies

Recent evidence indicates that TRAIL-based therapies intersect with immune checkpoint pathways, particularly PD-L1. Checkpoint blockade may potentiate TRAIL efficacy through two complementary mechanisms. First, tumor-intrinsic resensitization occurs when PD-L1 inhibition disrupts survival pathways that blunt TRAIL signaling. Second, immune-mediated enhancement arises from relieving PD-1/PD-L1-dependent suppression of cytotoxic T cells, thereby increasing immune pressure on tumor cells and complementing TRAIL’s ability to selectively eliminate transformed cells. Together, these effects position PD-L1/PD-1 inhibitors as rational partners for TRAIL-based therapies.

In triple-negative breast cancer, PD-L1 blockade (with siRNA targeting ERK or with the MAPK kinase inhibitor U0126) significantly increases TRAIL-mediated apoptosis [92]. In gastric cancer, dual targeting of PD-L1 (using miR-429 Mimics) and EGFR further amplifies apoptotic signaling and reduces tumor viability [93]. In renal cell carcinoma, co-delivery of the PD-L1 inhibitor ARB-272572 within TRAIL-loaded extracellular vesicles produced a potent combinatorial effect, overcoming TRAIL resistance and synergistically enhancing apoptosis in both in vitro and in vivo settings [195].

### 6.6. Targeting Metastatic Pathways to Restore TRAIL Sensitivity

Intratumoral administration of adenovirus vectors co-expressing human endostatin and soluble TRAIL produces a markedly stronger antitumor effect than adenoviral TRAIL alone, demonstrating that anti-angiogenic modulation can significantly enhance TRAIL-mediated tumor regression [196]. A similar anti-metastatic benefit was observed with immunotherapy combining CpG oligonucleotides and TRAIL delivered via adenoviral transduction, which effectively eliminated metastatic lung lesions in a murine renal cell carcinoma model through a T-cell-dependent mechanism [197].

Combining TRAIL with the Sphingosine kinase 1 (SPHK1) inhibitor PF-543 overcame acquired TRAIL resistance by synergistically enhancing apoptosis through activation of the mitochondrial pathway and modulating DcR1 and DR5 via the SPHK1/S1PR1/STAT3 axis. This combination also reduced cell aggressiveness and stemness, indicating that targeting SPHK1 may be an effective strategy to counter TRAIL resistance and limit metastasis in colorectal cancer cells [198].

## 7. Strategic Principles for Designing and Personalizing TRAIL Combination Therapies

### 7.1. Methods to Screen for Responders Before Treatment

To avoid exposing resistant patients to treatment-related toxicities without clinical benefit, screening methodologies must evaluate baseline molecular profiles. Immunohistochemistry can be used to evaluate baseline protein expression of DR4/DR5 [199] and glycosylation enzymes such as GALNT14 [100] in formalin-fixed, paraffin-embedded tumor tissue. ^89^Zr-CTB006 positron emission tomography (PET) was successfully used for noninvasive imaging of DR5 expression in preclinical models and patients with gastrointestinal cancers [200].

RNA sequencing of tumor biopsies can assess gene signature expression levels [96]. Functional assays to measure viability and apoptotic cleavage in vivo could be used before and alongside imaging to assess tumor regression. In mice, dual luciferase imaging was used to measure tumor regression and apoptosis by caspase activity [201]. Caspase 8 can be used as a functional predictive biomarker, for example, in 3D spheroid assays [201]. The serum caspase-3/7 levels were significantly increased in patients who received dulanermin [202]. Fine-needle aspirate sampling was a practical method to measure caspase activity to assess the on-target pharmacodynamics of conatumumab preclinically [203].

### 7.2. Reported Combination Indices

The Combination Index (CI) is a quantitative measure used to assess the type of drug–drug interaction (synergism, additivity, or antagonism). The most widely used method to calculate the CI is the Chou–Talalay method [204], which is based on the median-effect principle of the mass-action law. In this method, a CI value < 1.0 indicates synergy. Another method is that of Jin [205], based on the observed fractional effects of the drugs. In this method, a CI value > 1.15 indicates synergy. Despite numerous reports in the literature, very few of the studies cited above provide a detailed investigation of the synergy between TRAIL and other compounds used in combinatorial therapy across a range of doses. Table 3 summarizes reports from the literature that present values of combination indices, reflecting treatment efficacy. Data indicate that the dependence of the synergistic effects on the concentrations of the therapeutic agents is complex and, in certain cases, occurs within a therapeutic window. Thus, protocols should be optimized to ensure maximal synergy between the drugs.

### 7.3. Tumor-Type-Specific Guidance for TRAIL Combinatorial Therapies

A wide range of combinations was tested across multiple cell types, covering a broad spectrum of cancers. The rationale for selecting these combinations has been outlined earlier, but it is worth emphasizing that their preferential activity in specific malignancies reflects the context-dependent modulation of DR4/DR5 and decoy receptors by partner drugs, the intrinsic apoptotic rheostat, and tumor-subtype-specific vulnerabilities.

The sensitivity of the various cell lines to TRAIL was cross-validated with the comprehensive dataset published by Nair et al. [61], and IC_50_ values for TRAIL were included whenever available. A systematic comparison linking each tumor type to its most representative TRAIL-based combinations is provided in the Supplemental Table S1.

## 8. Limitations of TRAIL-Based Combinatorial Approaches

Despite nearly three decades of intensive research on TRAIL and the development of rational strategies to enhance its apoptotic activity, these approaches have not yet entered routine clinical use. The main obstacles are: poor concordance between in vitro and in vivo responses, the scarcity of predictive biomarkers, pharmacokinetic limitations of TRAIL agonists, safety constraints, limited durability of response, and the complex regulatory requirements for biologic combinations.

Many TRAIL combinations show strong synergy in vitro but rarely translate into meaningful antitumor activity in vivo. Cell line models typically overexpress DR4/DR5, lack stromal and immune components, and do not reproduce the spatial, metabolic, and phenotypic heterogeneity of human tumors, in which receptor expression is patchy, dynamically regulated, or suppressed by the microenvironment [206]. The tumor microenvironment, comprising both cellular components (fibroblasts, endothelial, myeloid, and lymphoid cells) and non-cellular components (extracellular matrix, mechanical forces, acidity, and hypoxia), interferes with TRAIL signaling [207]. Newer platforms that better mimic this context, such as patient-derived organoids, give more realistic results: in a clinically relevant organoid model, a more-than-additive effect was observed between the second-generation TRAIL receptor agonist APG 880 and radiation [208].

This gap directly raises the pharmacokinetic bar. Co-administered drugs may have mismatched pharmacokinetics or alter each other’s hepatic metabolism, protein binding, or biodistribution, making it unlikely that both agents reach effective concentrations at the tumor simultaneously [209]. TRAIL agonists have short serum half-lives and act rapidly, whereas many sensitizers require sustained pathway modulation, so sequencing matters more than co-delivery. Early clinical studies showed that recombinant TRAIL doses from 0.5 to 30 mg/kg were safe and reached plasma concentrations comparable to preclinical studies [58]. Still, priming is not universally beneficial, since some priming agents also upregulate decoy receptors and blunt TRAIL’s effect [210]. This creates a narrow window of opportunity: TRAIL given too early meets a refractory apoptotic machinery, while given too late, compensatory survival pathways (such as NF-κB or Akt) may have rebounded.

These dosing constraints in turn limit how aggressively combinations can be used. Although TRAIL agonists generally have a favorable therapeutic index, combination regimens raise concerns about off-target and synergistic toxicity. In trials combining dulanermin with paclitaxel/carboplatin in NSCLC, most adverse events were attributed to the cytotoxic backbone and noted transient hepatotoxicity [202]. TRAIL combined with HDAC inhibitors caused hepatotoxicity in tissue from patients with liver steatosis or hepatitis C [211], tagged recombinant TRAIL variants proved more hepatotoxic than native ones [212], and TRAIL plus bortezomib caused severe hepatotoxicity alongside cancer-specific apoptosis within a narrow therapeutic window [213]. A trial of mapatumumab in combination with paclitaxel and carboplatin in advanced NSCLC showed no benefit [214].

Even when a safe, synergistic regimen is achieved, responses are often not durable. Cancer stem cells frequently resist TRAIL through upregulation of c-FLIP [49] and Bcl-2 [48], so regimens that fail to co-target them are followed by recurrence. Prolonged exposure also drives acquired resistance through receptor internalization [215], shedding [216], decoy receptor upregulation [217], and compensatory NF-κB [218] or PI3K/Akt [97] signaling. Long-term data remain scarce. Animal studies are encouraging, with 65% inhibition of tumor growth and improved survival in colon cancer xenografts [5] and an 87% reduction in primary bone tumor growth in Ewing’s sarcoma and osteosarcoma models [219]. However, most xenograft studies use short-term tumor volume as the endpoint and miss late recurrence. In patients, a phase II study of conatumumab plus FOLFIRI in KRAS-mutant metastatic colorectal cancer showed only modest gains: progression-free survival of 6.5 versus 4.6 months and overall survival of 12.3 versus 12.0 months [220].

Finally, these accumulated uncertainties translate into stricter regulatory requirements. Combinations involving biologics require a clear mechanistic rationale, dedicated combination-specific toxicology, and evidence that each component contributes meaningfully to clinical benefit, including data on immune activation, hepatotoxicity, cytokine release, and drug–drug interactions.

## 9. Future Perspectives

Several priorities can be pursued with tools and agents already available. Predictive biomarkers need to be validated in clinical cohorts, not only in cell line panels. Combinations that act through ER stress and CHOP signaling deserve priority in early-phase testing, since this mechanism currently has the broadest and most consistent preclinical support. Preclinical work should move toward more physiologically relevant models, such as patient-derived organoids. Dosing schedules should follow the underlying mechanism rather than convention, so that TRAIL is given when receptor availability is highest.

Other priorities will require further technological or clinical development. Next-generation TRAIL agonists and delivery systems are needed to achieve better pharmacokinetics and tumor selectivity. Combination regimens should also target cancer stem cells, and not only shrink the bulk of the tumor, since durability depends on eliminating these resistant populations. TRAIL and immune checkpoint inhibitor combinations should be tested in additional tumor types, building on the consistent results already seen in breast, gastric, and renal cell carcinoma. Regulatory frameworks adapted to biologic combinations also need to be established, informed by data from completed and ongoing trials.

These problems are best addressed in sequence rather than all at once. That is the most realistic route to finally turning the preclinical promise of TRAIL-based combinations into routine clinical use.

## 10. Conclusions

TRAIL induces apoptosis in a wide range of transformed and tumor cells while largely sparing normal cells, but its initial therapeutic promise has been limited by two persistent problems: the poor pharmacokinetics of recombinant TRAIL and the intrinsic or acquired resistance of many cancers to TRAIL-induced apoptosis. These limitations prompted the development of combinatorial strategies aimed either at sensitizing resistant cells or at synergizing with TRAIL to trigger apoptosis.

The combinatorial strategies explored to date span a broad conceptual spectrum. Some rely on empirical observations of natural compounds that modulate apoptosis, whereas others follow a rational design approach, including targeted TRAIL delivery or inhibition/silencing of defined molecular resistance nodes. A deeper mechanistic understanding of TRAIL signaling and resistance is essential for advancing these rational approaches and for identifying therapeutic targets. Two points deserve emphasis. First, the success of combinatorial treatments explored to date is highly tumor-type-specific, so results in one cancer type cannot be assumed to generalize to another. Second, the mechanistic nodes reviewed here differ considerably in how well they are currently supported: death receptor induction through ER stress signaling is the most consistently documented, whereas decoy receptor suppression, though mechanistically distinct and potentially valuable, is supported by far fewer studies. Broad generalizations remain premature; the collective evidence reviewed here suggests that combining TRAIL with selected therapeutic agents may be a promising avenue for overcoming resistance and restoring apoptotic competence in cancer cells.

## Figures and Tables

**Table 1:** The main features of TRAIL isoforms.

Isoform	Structure	Secretion	Functionality	Apoptosis	Receptor Interactions
TRAIL-α	Full-length, transmembrane form	Cleavable	Strongly active	Robust induction	Binds DR4/5 efficiently
TRAIL-β	Lacks transmembrane domain	Secreted	Variable	Moderate induction	Binds DR4/5, weaker clusters
TRAIL-γ	Truncated extracellular domain	Secreted	Weak/ nonexistent	Minimal impact	Binds poorly DR4/5, DcR1/2

**Table 2:** TRAIL receptors involved in canonical TRAIL signaling.

Receptor	Oligomerization	Structural Features	Signaling Outcome	Refs.
DR4	Homotrimer Heterotrimer with DR5	Type I transmembrane full death domain; forms DISC	Apoptosis	[20,21]
DR5	Homotrimer Heterotrimer with DR4	Type I transmembrane full death domain; forms DISC	Apoptosis	[20,21]
DcR1	Heterotrimers with DR5	GPI-anchored; lacks transmembrane and intracellular domains	Blocks DISC assembly	[24,25,26]
DcR2	Heterotrimers with DR5	Truncated death domain; can trimerize but cannot recruit FADD	Blocks DISC assembly	[27,28,29]
OPG	Homodimers	Soluble decoy; binds TRAIL in circulation	Sequesters TRAIL	[30,31,32]

**Table 3:** Compounds synergistic with TRAIL with reported combination indices (CI). The values were calculated based on the Chou–Talalay algorithm, where CI < 1.0 indicates synergy [204], except for periplocin [129], calculated with Jin’s algorithm, where CI > 1.15 indicates synergy [205].

Compound	Experimental Model	Doses and CIs	Observations	Refs.
Dihydro- artemisinin	BxPC-3, PANC1 xenografts	DHA 25–100 μM; TRAIL 100 ng/mL CI < 0.9	Increased synergy with increasing doses	[118]
Bortezomib	Burkitt’s lymphoma	bortezomib: 6.25–200 nM TRAIL 100 ng/mL, Raji cells: CI: 0.04971–0.12527 CA46 cells: CI: 0.24680–0.08510	Raji cells: U-shaped dependence of CI on bortezomib dose; CA46 cells: Synergy decreases with increased bortezomib dose	[125]
Periplocin	esophageal squamous cell carcinoma in vitro	Periplocin: 50–200 ng/mL TRAIL: 1 μg/mL YES-2: CI: 1.26–1.16 KYSE-150: CI: 1.28–1.19 KYSE-510: CI: 2.09–1.38	Synergy at the lowest periplocin doses in YES-2, KYSE-150; at higher doses, the effect is rather additive; in KYSE-510, synergy decreases with higher periplocin doses and plateaus	[129]
IL-6	SKBR3 breast cancer cell line	IL-6: 0.2–12.5 ng/mL TRAIL: 0.2–25 ng/mL, CI: 0.45		[135]
Colchicine	MDA-MB-231, HCT116, H460	Colchicine 100 ng/mL TRAIL 5–20 ng/mL: MDA-MB-231: CI: 0.230–0.538 TRAIL 1.25–5 ng/mL: HCT116: CI: 0.099–0.297 TRAIL 5–20 ng/mL: H460: CI: 0.618–0.533	MDA-MB-231 and HCT116: synergy decreases with increasing TRAIL doses; H460: synergy increases with increasing TRAIL doses	[138]
Trifluorothymidine	NSCLC	TFT 5 μM TRAIL 150 ng/mL: A549: CI: 0.6 ± 0.2 TRAIL 10 ng/mL: H460: CI: 0.6 ± 0.2	Successive treatment with TFT + TRAIL and TFT alone was synergistic. Successive treatment with TFT and TFT + TRAIL was antagonistic.	[142]
Obatoclax	NSCLC	Obatoclax: 0.01–1 μM TRAIL 1 ng/mL: H460: CI: 0.63 ± 0.1 TRAIL 50 ng/mL: A549: CI: 0.73 ± 0.1 TRAIL 50ng/mL: SW1573: CI: 0.04 ± 0.01		[149]
Decursin	NSCLC	Decursin 20–100 μM TRAIL 20 ng/mL CI < 0.8	A549: synergy increases with increasing decursin doses; H596, Calu-1: synergy independent of decursin doses; H1299: U-shaped dependence on decursin doses	[150]
Sialic acid-binding lectin	malignant mesothelioma	TRAIL 1 ng/mL, SBL 0.5 μM, CI: 0.6		[157]
IFN-α	renal cell carcinoma	IFNα: 250–2000 ng/mL TRAIL: 62.5–500 ng/mL A498: CI: 0.255–0.620 ACHN: CI: 0.825–0.525	The CIs vary nonlinearly. The synergistic effect occurs in a defined therapeutic window	[167]
Sorafenib	8505C thyroid cancer cell line	Sorafenib 32 μM TRAIL 25 ng/mL: CI: 0.365 TRAIL 250 ng/mL: CI: 0.38		[169]
Thapsigargin	Esophageal squamous cell carcinoma	Thapsigargin 1–2 μM TRAIL 100–200 ng/mL EC109: CI: 0.84; TE12: CI: 0.80		[174]
CPT-11	C4-2 cells	CPT-11: 2–200 ng/mL TRAIL 10–1000 ng/mL CI: 0.096–0.059	Highly synergic	[184]

## Data Availability

No new data were created or analyzed in this study. Data sharing is not applicable to this article.

## Supplementary Materials

The following supporting information can be downloaded at: https://www.mdpi.com/article/10.3390/ijms27167068/s1.

## Abbreviations

The following abbreviations are used in this manuscript:

AML	Acute Myeloid Leukemia
Apaf-1	Apoptotic protease-activating factor 1
Apo2L	Apoptosis Antigen 2 Ligand
Apo2L.XL	Cross-linked Apo2 Ligand
Atf4	Activating Transcription Factor 4
ATP	Adenosine triphosphate
Bad	Bcl-2-associated agonist of cell death
Bak	Bcl-2 antagonist/killer 1
Bax	Bcl-2-associated X protein
Bcl-2	B-cell lymphoma 2
Bcl-x	B-cell lymphoma-x
Bcl-xL	B-cell lymphoma-extra large
CDK	Cyclin-dependent kinase
CHOP	C/EBP Homologous Protein
cIAP	Cellular inhibitor of apoptosis protein
CK2	Casein kinase 2
CML	Chronic Myeloid Leukemia
CpG	Cytosine–phosphate–guanine
CRISPR	Clustered Regularly Interspaced Short Palindromic Repeats
c-FLIP	Cellular FADD-like interleukin-1-converting enzyme inhibitory protein
c-FLIP(L)	Cellular FADD-like interleukin-1-converting enzyme inhibitory protein, long isoform
DcR1	Decoy Receptor 1
DcR2	Decoy Receptor 2
DR4	Death Receptor 4
DR5	Death Receptor 5
DISC	Death-Inducing Signaling Complex
DHA	Dihydroartemisinin
EGCG	Epigallocatechin-3-gallate
EGFR	Epidermal Growth Factor Receptor
ERK	Extracellular Signal-Regulated Kinase
FADD	Fas-associated protein with a death domain
FasL	Fas ligand
FLICE	FADD-like IL-1β-converting enzyme
FLIP	FADD-like inhibitor protein
Fn14	Fibroblast growth factor-inducible 14
GBM	Glioblastoma multiforme
GPI	Glycosylphosphatidylinositol
GSK-3β	Glycogen Synthase Kinase 3 Beta
HDAC	Histone deacetylases
HDACi	Histone deacetylase inhibitor
IAP	Inhibitor of apoptosis protein
IFN	Interferon
IKK	IkB kinase
JAK2	Janus Kinase 2
JNK	c-Jun N-terminal kinase
MAPK	Mitogen-activated Protein Kinase
Mcl-1	Myeloid cell leukemia-1
mDR5	Murine death receptor 5
MEK1/2	Mitogen-activated protein kinase 1/2
MG-132	Z-Leu-Leu-Leu-al
miR	microRNA
mRNA	Messenger ribonucleic acid
MSC	Mesenchymal stromal cell
mTOR	Mechanistic Target of Rapamycin
NF-kB	Nuclear Factor kappa-light-chain-enhancer of activated B cells
OPG	Osteoprotegerin
PD-L1	Programmed Death-Ligand 1
PERK	Protein kinase RNA-like ER kinase
PED/PEA	Phosphoprotein Enriched in Diabetes/Phosphoprotein Enriched in Astrocytes
PI3K	Phosphoinositide 3-kinase
PIM	Proviral Integration site for Moloney murine leukemia virus
PPAR	Peroxisome Proliferator-Activated Receptor
p-STAT3	Phospho-Signal Transducer and Activator of Transcription 3
RIP	Receptor-interacting protein
rhTRAIL	Recombinant human TRAIL
RIPK-1	Receptor-Interacting Protein Kinase 1
ROCK2	Rho-associated coiled-coil forming protein kinase 2
ROS	Reactive Oxygen Species
S1PR1	Sphingosine-1-phosphate receptor 1
SBHA	Suberohydroxamic acid
scTRAIL	Single-chain TRAIL
SHP-1	Src homology region 2 domain-containing phosphatase-1
shRNA	Short hairpin RNA
siRNA	Small interfering RNA
Smac	Second Mitochondria-derived Activator of Caspases
SPHK1	Sphingosine kinase 1
STAT3	Signal Transducer and Activator of Transcription 3
SQSTM1	Sequestosome 1
tBid	Truncated BH3 interacting-domain death agonist
TQ	Thymoquinone
TRAIL	Tumor Necrosis Factor (TNF)-Related Apoptosis-Inducing Ligand
TNF	Tumor necrosis factor
TNFSF10	TNF superfamily member 10
TNBC	Triple-Negative Breast Cancer
TP53INP2	Tumor Protein p53 Inducible Nuclear Protein 2
TRAIL-R	TRAIL receptor
TWEAK	Tumor necrosis factor-like weak inducer of apoptosis
