# Supplementary material for: Helping Apo2L/TRAIL in the Battle: Synergistic Therapeutic Approaches for Cancer Therapies
Source: Int J Mol Sci. 2026 Aug 7;27(16):7068. doi: 10.3390/ijms27167068 (PMC13512678; doi:10.3390/ijms27167068)
Supplement: Supplementary file 1 [file ijms-27-07068-s001.zip › ijms-4450707-supplementary.pdf]

Supplemental Table S1. Tumor-specific TRAIL combinations and mechanistic rationale. IC50 values for TRAIL resistance, when available in [61], were added as superscripts to the corresponding cell lines. When IC50 data were not reported, cell lines were classified as R (resistant) or S (sensitive) to TRAIL based on the authors' evaluation

Lineage -based cancers	Cancer Type	Cell Line$^{IC50, R/S}$	Molecular Mechanisms	Adjuvant Treatment	Ref.
Hematologic	non-Hodgkin's B-lymphoma	VAL^{R}, RL^{R}	↓Mcl-1	Quercetin	[110]
		Raji^{R}	↑ DR5, ↑ MAPK	Bortezomib	[125]
		B-NHL^{R}	YY1 inhibition	Rituximab	[183]
		Jurkat^{3}	↓P-STAT3, ↓cIAP-1/2	AG-490	[170]
	CML	K562^{R}	↓XIAP, ↓FLIP	Hydroxychavicol	[153]
	AML	OCI-AML3^{3}	Bcl-2 inhibitor	Venetoclax	[154]
Neuro-ectodermal tumor	Glio-blastoma	U251MG^{R}, U87MG^{R}	↑DR5; ↓c-FLIP;	Monensin	[114]
		T98G^{3}	↑DR5	Salinomycin	[115]
		U87MG^{R},U87MG-R50^{R},U373^{R}	↑DR4/DR5	Mitoxantrone	[141]
		U-138MG^{R}	↑DR5	IFN-β	[137]
		U87^{R}, A172^{S}, T98G^{S}	↑DR5, ↓DR4,↑ NF-κB	miR-133a	[131]
		MZ304^{R}, U343^{R}	IAP antagonist + Bcl-2 inhibitor	Birinapant + ABT-199	[155]
		U87MG^{R}	↑DR5	SGI-1776	[172]
		U87^{R}, U138^{R}	↑Bax, ↓FLIP, XIAP, Bcl-2	Compound C	[173]
		LN229^{3}, U251^{R}	↑DR4/DR5	MS-275	[192]
		U87MG^{R}, U373^{R}, T98G^{3}	↑ROS, ↑HMOX	Chaetocin	[193]
	Medullo-blastoma	Medulloblastoma cells	↑caspase-8	Valproic + IFNγ	[189]
	Melanoma	A375^{3}	↑DR4/DR5	EGCG, Quercetin	[121]
		A375^{3}, SK-MEL-28^{3} G361$^{0.25}$, CRL1579^{R}, MeWo^{3}	↑DR4/5, ↑caspase-3, -8, -9	Interferon β	[136]
		Primary human melanoma cells	↓c-FLIP$_{L/S}$, ↑caspase-8, ↓c-IAP1, c-IAP2, XIAP	ZD6244 BEZ235	[165]
Mesenchymal & mesenchymal -like tumors	Osteo-sarcoma	U2-OS^{3}, MG-63^{3}	↑caspase-8	ROCK2 silencing	[171]
	Mesothelioma	NCI-H28$^{0.01}$	↑caspase 8,-9,-3, ↑ tBid	Lectin (*Rana catesbeiana*)	[157]
		MM-BI, Ist-Mes2	HDAC inhibitor	SBHA	[190]
Epithelial – Adenocarcinoma	Breast cancer	MDA-231^{3}	↓PD-L1	U0126	[92]
		MDA-MB-231^{3}, MCF-7^{3}	↑DR5 via ROS, ↓IAP	Curcumin	[117]
		BT-20^{3}, MCF-7^{3}	↑DR5, ↓c-FLIPL	Quercetin	[109]
		MDA-MB-231^{3}	↑DR5	Theophylline	[146]
		MCF-7^{3}, MDA-MB-231^{3}	G1 phase cell cycle arrest	Daurisoline	[145]
		MDA-MB-231^{3}	↑ DR5, ↑ Caspase 3	Colchicine	[138]
		MDA-MB-231^{3}, MCF-7^{3}, T47D^{R}	↑DR4/DR5 ↑Bad, Bax, ↑caspase-3, -8,	Chemo-therapeutics	[139]
		MDA-MB-231^{3}, MCF-7^{3}	↑DR4/DR5	Thymoquinone	[143, 144]
		SKBR3^{3}	↑ DR 4/5, ↑ Caspase 3,8,9	IL-6	[135]
		MDA-MB-231^{3}, SK-MEL-28	↓Akt activity, ↑RhoA, ↑	Statins	[166]

Epithelial – Adenocarcinoma	Endometrial cancer	Hec50co^{R}	↓XIAP	LBH589 (HDACi)	[191]
	Gastric cancer	BGC823^{R}, SGC7901^{R}	↓PD-L1	miR-429 Mimics	[93]
	Colon and colorectal cancer	Caco-2^{3}	↑DR4/DR5	EGCG	[119]
			↑DR5 via ROS/ER stress;	artesunate	[113]
			↑DR4	CRISPR (↑Klotho gene)	[130]
		LS174T^{3}, LS180^{3}, HCT116$^{0.04}$, CX-1^{S}	↑JNK-Bcl-xL-Bak pathway ↑ caspase 8, 9 and 3	mitomycin C, hyperthermia	[158]
		SW620^{3}	↓AKT, ↓NF-kB, ↑CHOP	Metformin	[178]
		HCT116$^{0.04}$	↑ DR5, ↑ Caspase 3	Colchicine	[138]
		HCT116$^{0.04}$	↓NF-κB → ↓Bcl-xL	Sulindac sulfide/ IKK peptide	[176]
		HCT116-TR^{R}	↓DcR1; ↑DR5 via SPHK1/S1PR1/STAT3	PF-543	[198]
		HT-29^{3}	↓cFLIP, Mitochondria	Brequinar	[186]
		SW480^{3}	↑ caspase-8	Trichostatin A	[187]
	Lung cancer	A549^{3}	↑DR4/5; ↑Bad, ↑Bax; ↓Bcl-x, ↓Bcl-2, ↓c-FLIP	Apigenin	[127] [128]
		A549^{3}, H596^{R}, H1299^{R}	↑DR5, ↑CHOP,	Tanshinone IIA	[112]
		A549^{3,} H460^{S}	↑DR5, ↓cFLIP, ↓XIAP, ↑Bax	Trifluoro-thymidine	[142]
		NCI-H460^{S}, A549^{3}	↓DcR2; ↑caspase-8/9/Bax	Cisplatin	[132]
		A549^{3}, SW-1573^{3}	↑DR5; ↓XIAP	Obatoclax	[149]
		A549^{3}, H596^{R}, H1299^{3}, Calu-1^{R}	↑ROS, ↑PERK/ATF4 ↑DR5, ↓survivin, ↓Bcl-xL	Decursin	[150]
		A549^{3}	↑DR5, ↑ROS,↑PERK/ATF4,	AdV IKKβKA	[177]
		U1690^{R}	IKK inhibition	Doxorubicin, brequinar	[186]
	Ovarian cancer	SKOV-3^{R}, TOV-21G^{3}, OVCAR-3^{S}, HOSE^{S}	↑DR5, ↑CHOP, ↑ER stress	Quercetin	[107]
		COV434^{3}, KGN^{R}	↑DR5, ↑Bax	Cisplatin	[148]
	Pancreatic cancer	MIA PaCa-2$^{0.06}$	↓Bid	EGCG	[124]
			↑ROS, ↑ DR5,↑ Caspase-8	Dihydro-artemisinin	[118]
		PANC-1^{3}	↓CDK; ↓XIAP/Mcl-1/Bcl-2	ZD55-TRAIL - Smac + SNS-032	[159]
		Panc89, Colo357	Cell cycle arrest	CDK9 inhibitor- Atuveciclib	[160]
		PANC-1^{3}, AsPC-1^{3}	↑DR4, ↓histone methylation	Chaetospiro-lactone	[194]

Epithelial – Adenocarcinoma	Prostate cancer	PC-3[3]	↑DR5	Apigenin	[126]
			↑DR4/DR5	Ursolic acid	[111]
			↑DR5; CHOP-dependent	Tunicamycin	[116]
			↑DR4/5	Paclitaxel	[140]
			↑DR5	Rosiglitazone	[182]
		LNCaP[R], DU145[3]	↑AMPK	Troglitazone	[179]
		LNCaP[3]	↑DR5, ↓β-catenin, ↓c-FLIP;	Rosiglitazone	[181]
		LNCaP clone FGC[3]	↓Akt activity, ↓c-FLIP$_{L/S}$	Matairesinol	[163]
		PC-3[3], LNCaP[R], Du145[3]	↓Akt	Triciribine	[164]
		PC-3[3]	↓p65-Ser536	CK2 inhibitor	[175]
		C4-2[R]	↑PCNA, ↑Caspase 3	Irinotecan	[184]
Epithelial – Hepatocellular	Hepatocellular carcinoma	HepG2[3]	↑DR4/5;	EGCG	[120]
		SMMC7721[3], Hep3B[R], BEL7404[R]	CDK inhibitor	ZD55-TRAIL + Smac + SNS-032	[161]
		HepG2[3]	↓XIAP, ↓cIAP-1, ↓Bcl-xL	J7	[151]
		Huh-7[3], Hep3B[R],	↓ p-STAT3	Dovitinib	[168]
		HepG2[3], Huh-7[3]	↓c-FLIP	Valproic acid / ITF2357	[188]
		HepG2-derived tumors	↑apoptosis, ↓angiogenesis	Endostatin (AdV)	[196]
Epithelial – Renal type	Renal carcinoma	786-O[3]	↓FLIP/Mcl-1 via ROS;	EGC5G	[123]
		Caki-1[0.09]	↑ROS, ↓cFLIP(L), ↓Mcl-1	Compound C	[152]
		Caki[R]	↑DR5,↑ROS; ↓c-FLIP;	Rosiglitazone	[182]
		A-498[S], ACHN[R], 786-O[3]	↑ERK, ↑caspase 8, -9, -3	IFNα	[167]
		Renca[R]	↑T-cell killing	CpG	[197]
		HeLa[3]	↓c-FLIP, ↓Survivin	YM155	[147]
		786-O[3], Caki-1[R], A498[S]	↓PD-L1	ARB-272572	[195]
	Thyroid cancer	8505C[R]	Jak2/Stat3, ↓Mcl1	Sorafenib	[169]
	Esophageal cancer	EC109[S], TE12[S]	↑DR5, ↑CHOP, ↑AMPK, ↑ROS	Thapsigargin	[174]
	Esophageal cancer	YES-2[R], KYSE-150[R], KYSE-510[R]	↑DR4/5 by ↓FoxP3 ↑Caspase3, ↑FADD,	Periplocin	[129]
	Naso-pharyngeal carcinoma	CNE-1[R], CNE-2[R], NP69[R], C666-1[R]	↓Bcl-XL, Bcl-2, FADD, FLIP, ↑caspase-3, -8, -9, ↓NF-κB	EGCG	[122]
Epithelial – Urothelial carcinoma	Bladder carcinoma	HT-1376[R] RT112[S], T24[S], 253J[S]	Bcl-2 inhibition	Gemcitabine	[156]
